# Trajectories of early secondary insults correlate to outcomes of traumatic brain injury: results from a large, single centre, observational study

**DOI:** 10.1186/s12873-018-0197-y

**Published:** 2018-12-05

**Authors:** Paola Cristina Volpi, Chiara Robba, Matteo Rota, Alessia Vargiolu, Giuseppe Citerio

**Affiliations:** 1 School of Medicine and Surgery, University of Milan-Bicocca, Via Cadore 48, 20900 Monza, MB Italy; 2 Neurocritical Care Unit, Addenbrooke’s Hospital, Cambridge University, Box 1, Addenbrooke’s Hospital, Cambridge University Hospitals Trust, Hills Road, Cambridge, CB2 0QQ UK; 3 Anaesthesia and Intensive Care, Policlinico San Martino IRCCS for Oncology, Largo Rosanna Benzi, 16132 Genoa, GE Italy; 4 Department of Clinical Sciences and Community Health, University of Milan, Milan, Italy; 5 Neurointensive Care Unit, San Gerardo Hospital, ASST-Monza, Via G. B. Pergolesi 33, 20835 Monza, MB Italy

**Keywords:** Traumatic brain injury, Secondary injuries, Prehospital insults, Trajectory, Outcome

## Abstract

**Background:**

Secondary insults (SI), such as hypotension, hypoxia, and intracranial hypertension frequently occur after traumatic brain injury (TBI), and have a strong impact on patients’ clinical outcomes. The aim of this study is to examine the trajectories of SI from the early phase of injury in the prehospital setting to hospital admission in a cohort of TBI patients.

**Methods:**

This is a retrospective, observational, single centre study on consecutive patients admitted from 1997 to 2016 to the Neuro Intensive Care Unit (NICU) at San Gerardo Hospital, in Monza, Italy. Trajectories of SI from the prehospital to hospital settings were defined as “sustained”, “resolved”, “new event”, and “none”. Univariate and multivariate logistic regression analyses were performed to correlate SI trajectories to a 6-months outcome.

**Results:**

Nine hundred sixty-seven patients were enrolled in the final analysis. About 20% had hypoxic or hypotensive events and 30.7% of patients had pupillary abnormalities. Hypotension and hypoxia were associated with an unfavourable outcome when “sustained” and “resolved”, while pupillary abnormalities were associated with a poor outcome when “sustained” and as “new events”. After adjusting for confounding factors, 6-month mortality strongly correlated with “sustained” hypotension (OR 11.25, 95% CI, 3.52–35.99), “sustained” pupillary abnormalities (OR 2.8, 95% CI, 1.51–5.2) and “new event” pupillary abnormalities (OR 2.8, 95% CI, 1.16–6.76).

**Conclusions:**

After TBI, sustained hypotension and pupillary abnormalities are important determinants for patients’ outcomes. Early trajectories define the dynamics of SI and contribute to a better understanding of how early recognition and treatments in emergency settings could impact on 6-month outcomes and mortality.

## Background

Traumatic Brain Injury (TBI) is one of the leading causes of death and disability worldwide [1]. The mortality rate is 35% and about 55–60% of survivors suffer permanent neurological impairment, with a peak in the younger population and with consequent dramatic loss of productive life-years in the affected individuals [2]. It is well known that secondary insults (SI), such as hypoxia, hypotension, and intracranial hypertension in the early post-traumatic phase have a negative impact on patients’ outcomes [3–5]. In the International Mission for Prognosis and Analysis of Clinical Trials in Traumatic Brain Injury (IMPACT-TBI) study [3, 4], secondary insults were strongly associated with an unfavourable outcome (Odds Ratio, OR, 2.1 for hypoxia and 2.7 for hypotension), with the poorest outcome when two insults were concomitant; therefore, the Brain Trauma Foundation guidelines emphasize the importance of aggressive prevention and treatment of hypotension and hypoxia [6].

Previous studies evaluated dichotomically the presence or absence of secondary insults [7], without deeply exploring the effects of their evolution during the early phases of TBI, especially in the pre-hospital setting. This aspect is pivotal, since pathophysiological events are dynamic and could be potentially reversed by early treatment. Defining the trajectories of secondary insults, i.e. resolution, persistence, and new development, from the scene of the accident to hospital admission, could better describe their effects on patients’ outcomes and influence the decision-making process of early treatment, both in the pre-hospital setting and in the emergency department.

The aim of this study was to examine the trajectories of secondary insults in terms of incidence and long-term effects on 6-month neurological outcomes in a large cohort of consecutive TBI patients.

## Materials and methods

### Study design

This is a single centre, retrospective, observational study including all consecutive TBI patients admitted to the Neuro Intensive Care Unit (NICU) at San Gerardo Hospital, Monza, Italy from January 1st, 1997 to December 31st, 2016. Ethical requirements were fulfilled, accordingly to “Decreto Legge 196”, article 4 (2003). Due to the retrospective data analysis and the de-identification of sensible data, no consent was required for data utilization. Data were prospectively collected using a structured database, as previously described [8]. Patients aged > 18 years, admitted to NICU within 24 h after a TBI with any Glasgow Coma Scale (GCS) [9] were considered for inclusion in the analysis. Exclusion criteria were penetrating TBI, concomitant spinal cord lesions, TBI occurred more than 24 h before admission, and death before admission to NICU. Patients were treated as per the Advanced Life Support (ALS) and Advanced Trauma Life Support (ATLS) guidelines in the pre-hospital settings [10].

### Data collection

For each patient, we collected demographics, clinical characteristics as reported at the scene (age, sex, hour and date of trauma and hospital admission, clinical health state, pupillary reactivity and GCS, intubation and sedation), and data on the presence of extracranial lesions, including systemic bleeding, thoracic trauma, pneumothorax, abdominal trauma, limb trauma, facial trauma, pelvic fracture, spinal lesion and Computed Tomography (CT) scan at the admission, classified according to the Marshall score [11]. The assessment of clinical state before hospitalization was defined as good health, moderate or severe limitation to activity for chronic disease [12]. Data about the occurrence of secondary insults were collected at the scene, at hospital admission, and after stabilization. We considered the worst measurements recorded within the first hour at the scene and in the emergency department. Hypotension was defined as a systolic blood pressure (SBP) ≤ 90 mmHg, even in a single episode; hypoxia was defined as oxygen saturation (SpO_2_) ≤ 90%, even in a single episode; and pupillary abnormalities as signs of brain herniation and dramatic intracranial pressure were defined as “normal”, “bilaterally dilated, unreactive” and “anisocoric” (difference > 2 mm from contralateral one). Trajectories of secondary insults were defined as: “*Sustained”* when the insult was present in both the pre-hospital and hospital settings; *“Resolved”* when it was recorded in the pre-hospital setting, but not at hospital admission; *“New event”* when it was not documented in the pre-hospital setting, but only at the arrival at the emergency department; *“None”* when no insults occurred to the patient.

The primary outcome was to evaluate the incidence of trajectories of secondary insults from the pre-hospital setting to hospital admission; the secondary outcome was to correlate these trajectories to a 6-month neurological functional status. A follow-up at 6 months was conducted, by either a telephone interview or a clinical visit, using the Glasgow Outcome Scale (GOS), dichotomized into favourable (Good recovery/Moderate disability) and unfavourable outcome (Severe Disability/Vegetative/Death) [13].

### Statistical analysis

Categorical variables were reported as frequencies, continuous variable as median and range. The Chi-square test for categorical variables, or the Fisher’s exact test when expected frequencies were lower than 5, was used to compare extracranial lesions and early therapies across secondary insult trajectories. A smooth function was used to interpolate the occurrence of hypoxia, hypotension, pupillary abnormalities and age at trauma on the accident scene across year of trauma.

Univariate logistic regression was used to investigate the impact of trajectories of pupillary reactivity, hypoxia, hypotension, and their combination on 6-month outcome and mortality. Multivariate models aimed to identify the independent prognostic effect of trajectories of hypoxia, hypotension and pupillary abnormalities on 6-month unfavourable outcome and mortality were fitted after allowing for age, sex, intubation and sedation on scene, GCS, CT findings (Marshall score), presence of subarachnoid haemorrhage (SAH) and presence of any extracranial lesions. A complete-case analysis was carried out.

The performance of multivariable logistic regression model was assessed by computing the Area Under the Receiver Operating Characteristic Curve (AUC). An AUC of 0.50 indicates that the model has no discriminative power, while a model with an AUC of 1.0 reflects perfect discrimination.

Multivariable adjusted ORs and their 95% Confidence Intervals (CIs) were reported in a confidence-interval plot. The magnitude of SpO_2_ and mean arterial pressure (MAP) was compared across SI trajectories through the nonparametric Kruskal-Wallis test, using the Dunn’s test to account for multiple comparisons. Results were graphically represented through box-plots. A *p*-value< 0.05 was considered as significant. Statistical analyses were performed using SAS 9.4 (SAS Institute, Cary, NC) and R version 3.4.0 (R Foundation, Vienna, Austria) for graphs.

## Results

From 1997 to 2016, a total of 967 consecutive TBI patients admitted at the NICU of San Gerardo Hospital fulfilled the inclusion criteria and were included in the analysis (Table 1).

**Table 1:** Characteristics of TBI population admitted to San Gerardo Hospital NICU from January 1997 – till December 2016

Number TBI patients	967
Median age, years	45 (0–94)
Sex	
Female	240 (25%)
Male	727 (75%)
Pre-TBI functional state	
Good health	665 (69%)
Moderate limitation of activities	252 (26%)
Severe limitation of activities	43 (4%)
Missing	7 (1%)
Intubation at the scene	
No	435 (45%)
Yes	532 (55%)
Sedation at the scene	
No	464 (48%)
Yes	503 (52%)
GCS at the scene	
Mild TBI [13–15]	256 (27%)
Moderate TBI [9–12]	157 (16%)
Severe TBI [3–8]	554 (57%)
Intracranial lesions	
None	142 (15%)
Extradural haematoma	119 (12%)
Subdural haematoma	323 (33%)
Contusion	296 (31%)
Diffusion Axonal Injury/ Petechiae	87 (9%)
CT findings (Marshall Score)	
Marshall 1	75 (8%)
Marshall 2	370 (38%)
Marshall 3	75 (8%)
Marshall 4	21 (2%)
Marshall 5	365 (38%)
Marshall 6	61 (6%)
Extracranial lesions	
No	379 (39%)
Yes	588 (61%)

**Fig. 1:**
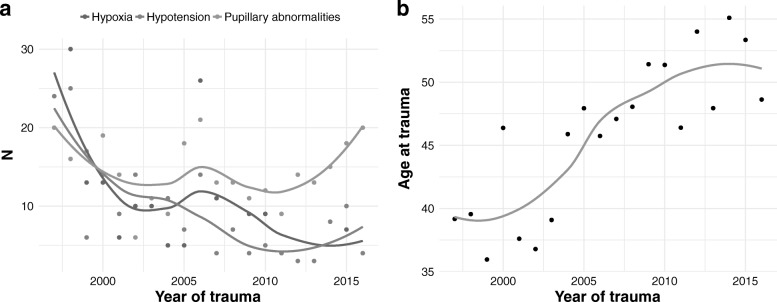
Smooth function of hypoxia, hypotension and pupillary abnormalities at the scene of the accident (panel a) and age at trauma (panel b) in TBI patients admitted during the study period

**Fig. 2:**
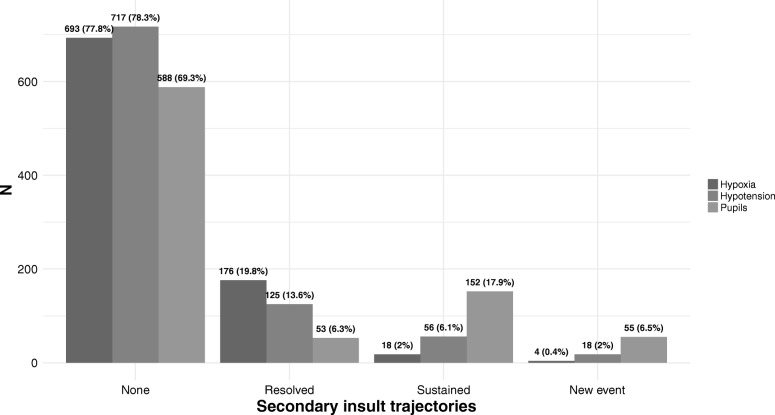
Distribution of trajectories of hypoxia, hypotension, and pupillary reactivity in TBI patients admitted to San Gerardo Hospital NICU during the study period

The mean age of the patients remarkably increased over the study period, while the prevalence of secondary injuries progressively decreased (Fig. 1a and b). About 80% of patients had no hypoxic or hypotensive events in the pre-hospital setting and at hospital admission, and 69.3% had no pupillary abnormalities (Fig. 2). Hypoxia was resolved at the admission in 19.8% of cases, was sustained in the 2%, and recorded as new event in 0.4% of the patients. Hypotension was resolved at the admission in 13.6% of cases, sustained in 6.1%, and observed as a new event in 2% of patients. Pupillary abnormalities were resolved at hospital admission in 6.3% of patients, sustained in 17.9%, and detected as a new event in 6.5%.

**Fig. 3:**
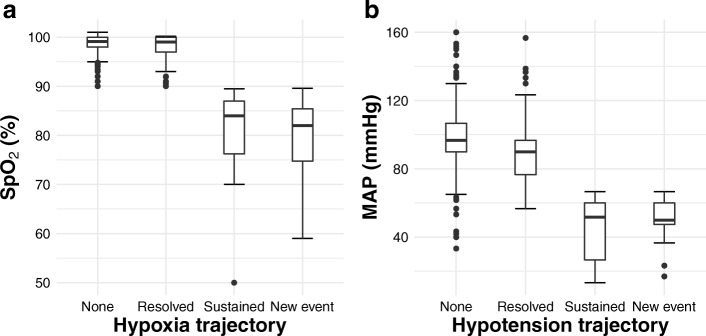
Differences in oxygenation (SpO_2_, Box Plot **a**) and mean arterial pressure (MAP, Box Plot **b**) across trajectories of hypoxia and hypotension. Box shows the interquartile range and the inner horizontal bold line indicates the median. Whiskers extend the median by ±1.5 times the interquartile range, while dotted points extending beyond the end of the whiskers represents outliers

Oxygenation, measured as SpO_2_, and MAP differed between patients without SI and those with resolved SI, although the difference was not clinically relevant (0.5% for SpO_2_ and 8.7 mmHg for MAP). No differences emerged between patients with sustained SI and those with new events; however, when patients were combined into two groups (none + resolved SI vs sustained + new events), a statistical difference was found (Fig. 3 a and b).

**Fig. 4:**
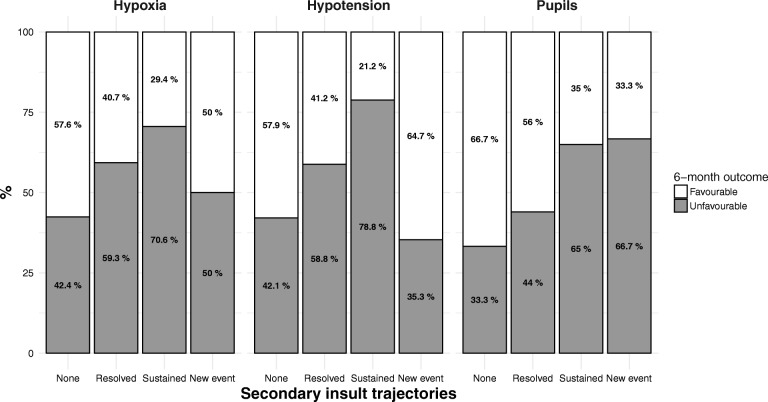
Distribution of 6-month outcome measured by GOS (4–5 = Favourable, 1–3 = Unfavourable) in trajectories of hypoxia, hypotension, and pupillary reactivity

At the 6-month follow-up, when hypoxia and hypotension occurred and were resolved at the admission to the emergency department, the rate of poor outcomes increased (59.3% vs 40.7% for hypoxia and 58.8% vs 41.2% for hypotension) (Fig. 4). When these secondary insults were sustained, the percentage of poor outcomes boosted up to 70.6% vs 29.4% in the hypoxia group, and to 78.8% vs 21.2% in the hypotension group. Pathological pupillary changes (sustained or new event) were associated with an unfavourable outcome (65% vs 35 and 66.7% vs 33.3% respectively), while normal pupils (no event or resolved) at admission were associated with a good outcome.

The relationship between trajectories of secondary injuries and outcomes are described in Table 2. Sustained hypoxia and sustained hypotension were associated with poor outcomes (OR 3.26, 95% CI 1.14–9.37, and OR 5.12, 95% CI 2.58–10.13) and mortality (OR 6.93, 95% CI 2.41–19.95, and OR 5.83, 95% CI 3.21–10.60). Pupillary changes were associated with poor outcome and mortality when sustained (OR 3.72, 95% CI 2.53–5.48, and OR 3.09, 95% CI 2.07–4.63), and when it occurred as a new event (OR 4.0, 95% CI 2.14–7.48, and OR 4.07, 95% CI 2.21–7.49).

**Table 2:** Univariate logistic regression analyses between trajectories of hypoxia, hypotension and pupils and 6-month outcomes (measured by GOS) or 6-month mortality

	6-month outcome				6-month mortality			
	Favourable	Unfavourable	OR (95% CI)	*P*-value	Survivors	Deaths	OR (95% CI)	*P*-value
Number of TBI patients^a^	480	415	–		637	258	–	
Hypoxia trajectory				< 0.01				< 0.01
None	374 (78%)	275 (66%)	1 (Reference)		482 (76%)	167 (65%)	1 (Reference)	
Resolved	61 (12%)	89 (21%)	1.98 (1.38–2.85)		91 (14%)	59 (23%)	1.87 (1.29–2.71)	
Sustained	5 (1%)	12 (3%)	3.26 (1.14–9.37)		5 (1%)	12 (4%)	6.93 (2.41–19.95)	
New event	2 (1%)	2 (1%)	1.36 (0.19–9.72)		4 (1%)	–	Not estimable	
Missing	38 (8%)	37 (9%)	–		55 (8%)	20 (8%)		
Hypotension trajectory				< 0.01				< 0.01
None	383 (80%)	279 (67%)	1 (Reference)		500 (78%)	162 (63%)	1 (Reference)	
Resolved	47 (10%)	67 (16%)	1.96 (1.31–2.93)		67 (11%)	47 (18%)	2.17 (1.43–3.27)	
Sustained	11 (2%)	41 (10%)	5.12 (2.58–10.13)		18 (3%)	34 (13%)	5.83 (3.21–10.60)	
New event	11 (2%)	6 (2%)	0.75 (0.27–20.5)		15 (2%)	2 (1%)	0.41 (0.09–1.82)	
Missing	28 (6%)	22 (5%)	–		37 (6%)	13 (5%)		
Pupils trajectory				< 0.01				< 0.01
None	360 (75%)	180 (43%)	1 (Reference)		447 (70%)	93 (36%)	1 (Reference)	
Resolved	28 (6%)	22 (5%)	1.57 (0.87–2.82)		40 (6%)	10 (4%)	1.20 (0.58–2.49)	
Sustained	50 (11%)	93 (23%)	3.72 (2.53–5.48)		87 (14%)	56 (22%)	3.09 (2.07–4.63)	
New event	16 (3%)	32 (8%)	4.00 (2.14–7.48)		26 (4%)	22 (8%)	4.07 (2.21–7.49)	
Missing	26 (5%)	88 (21%)	–		37 (6%)	77 (30%)		

^a^Six-months outcome was not available in 72 (7%) subjects

**Fig. 5:**
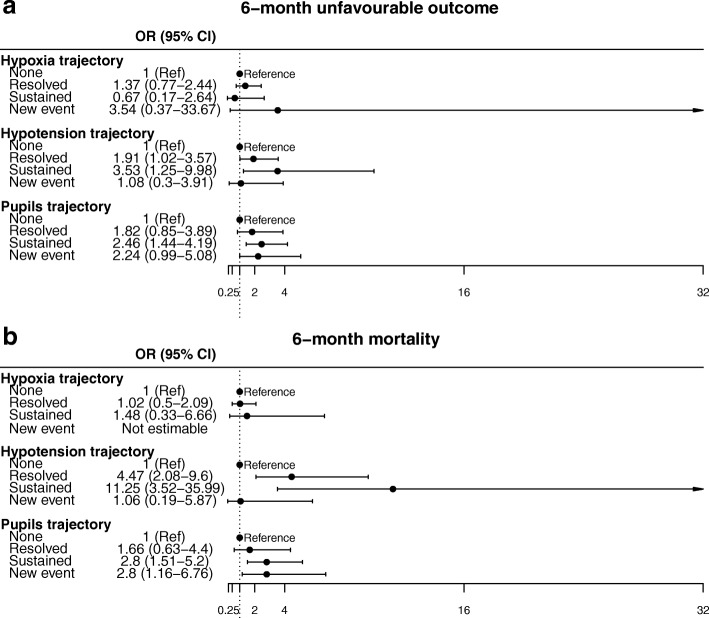
Confidence interval plot of trajectories of hypoxia, hypotension, and pupils in relation to 6-month functional state (**a**) and 6-month mortality (**b**). Horizontal lines represent the 95% conficence interval (CI). Odds Ratios (ORs) and 95% CIs were derived from multivariate logistic regression models, adjusted for age, sex, intubation on the scene, sedation on the scene, GCS at the ED, CT classification, presence of SAH, and extracranial lesions

In the multivariate logistic regression analysis, considering as covariates age, sex, intubation and sedation on scene, GCS, CT findings (Marshall score), presence of SAH and presence of any extracranial lesions, trajectories of hypotension were the strongest parameter associated with mortality (OR 11.25, 95% CI, 3.52–35.99) and unfavourable outcome (OR 3.53, 95% CI, 1.25–9.98), especially when sustained (Fig. 5). Trajectories of pupillary changes were the best neurological indicator for outcome and mortality with minimal data dispersion. AUCs were 0.84 for unfavourable outcome at 6 months and 0.89 for mortality, indicating a high discriminatory power of the model.

We also evaluated the association between trajectories and extracranial lesions. Only 1 and 3% of patients without extracranial injuries had persistent hypoxia and hypotension. Sixty-seven percent of patients with sustained hypoxia had the concomitant presence of chest trauma, while 61% of those with sustained hypotension presented with bleeding that required transfusions (Table 3). Early sedation and orotracheal intubation at the scene occurred in 64 and 82% of patients with resolved hypoxia, and among 69 and 81% of those with resolved hypotension, respectively. Interestingly, almost all patients (88%) with sustained hypotension were intubated at scene. Pupils’ trajectories had no relationship with extracranial lesions. However, a high proportion of patients with resolved (85 and 89%) and sustained (72 and 76%) insults were sedated and intubated at scene.

**Table 3:** Relationship between trajectories of hypoxia, hypotension, and pupils and extracranial lesions or therapies at the scene of the accident

	Extracranial lesions	Sedation at scene	Intubation at scene	Bleeding requiring transfusion	Chest trauma	PNX	Abdominal trauma	Limb trauma/ fractures	Facial trauma/ fracture	Pelvic trauma/ fractures	Amyelic spinal trauma
Hypoxia trajectory											
None (*n*=693)	374 (54%)	342 (49%)	335 (48%)	54 (8%)	152 (22%)	43 (6%)	57 (8%)	137 (20%)	188 (27%)	48 (7%)	18 (3%)
Resolved (*n*=176)	136 (77%)	112 (64%)	144 (82%)	41 (23%)	76 (43%)	24 (14%)	27 (15%)	70 (40%)	70 (40%)	25 (14%)	11 (6%)
Sustained (*n*=18)	16 (89%)	6 (33%)	10 (56%)	7 (39%)	12 (67%)	7 (39%)	3 (17%)	3 (17%)	3 (17%)	5 (28%)	1 (6%)
New event (*n*=4)	3 (75%)	2 (50%)	2 (50%)	0 (0%)	1 (25%)	1 (25%)	0 (0%)	2 (50%)	2 (50%)	0 (0%)	0 (0%)
*P*-value	< 0.01	< 0.01	< 0.01	< 0.01	< 0.01	< 0.01	0.02	0.11	< 0.01	< 0.01	0.08
Hypotension trajectory											
None (*n*=717)	396 (55%)	355 (50%)	344 (48%)	49 (7%)	153 (21%)	38 (5%)	43 (6%)	136 (19%)	209 (29%)	40 (6%)	15 (2%)
Resolved (*n*=125)	98 (78%)	86 (69%)	101 (81%)	26 (21%)	60 (48%)	15 (12%)	26 (21%)	41 (33%)	38 (30%)	25 (20%)	10 (8%)
Sustained (*n*=56)	50 (89%)	27 (48%)	49 (88%)	34 (61%)	29 (52%)	19 (34%)	15 (27%)	16 (29%)	24 (43%)	13 (23%)	5 (9%)
New event (*n*=18)	13 (72%)	9 (50%)	8 (44%)	1 (6%)	7 (39%)	1 (6%)	4 (22%)	5 (28%)	9 (50%)	4 (22%)	1 (6%)
*P*-value	< 0.01	< 0.01	< 0.01	< 0.01	< 0.01	< 0.01	< 0.01	0.03	0.05	< 0.01	< 0.01
Pupils trajectory											
None (*n*=588)	348 (59%)	264 (45%)	249 (42%)	53 (9%)	146 (25%)	44 (7%)	47 (8%)	130 (22%)	162 (28%)	48 (8%)	21 (4%)
Resolved (*n*=53)	32 (60%)	45 (85%)	47 (89%)	4 (8%)	20 (38%)	7 (13%)	7 (13%)	8 (15%)	17 (32%)	5 (9%)	2 (4%)
Sustained (*n*=152)	95 (63%)	109 (72%)	116 (76%)	21 (14%)	47 (31%)	12 (8%)	17 (11%)	32 (21%)	61 (40%)	14 (9%)	3 (2%)
New event (*n*=55)	24 (44%)	23 (42%)	27 (49%)	5 (9%)	5 (9%)	2 (4%)	1 (2%)	7 (13%)	12 (22%)	6 (11%)	1 (2%)
*P*-value	0.10	< 0.01	< 0.01	0.32	< 0.01	0.32	0.08	0.28	0.1	0.81	0.81

## Discussion

In this study, we describe the trajectories of secondary insults in patients with TBI and their effects on 6-month clinical outcomes. Over the last decades, the epidemiology and characteristics of TBI patients have been changing [14–18]: the TBI population is becoming older [19–21], the main mechanisms of trauma are more frequently accidental falls rather than road traffic accidents; the prevalence of secondary injuries is progressively decreasing. From 1997 to 2016, the TBI patients admitted in our institution showed a progressively lower rate of hypoxia and hypotension that dropped by 7% every year, likely due to different traumatic dynamics, increased age, and the prompt treatment of secondary insults.

By defining how secondary insults evolve from the pre-hospital setting to hospital admission-namely the early trajectories- we highlighted that the dynamics of secondary injuries could be an important parameter to provide insight into the therapeutic efficacy of first-aid procedures at the scene and in the emergency department and to improve clinical outcomes of patients after TBI.

Despite the fact that the harmful effect of secondary injuries on TBI patients’ outcomes has been previously described [22], little is known about the consequences of early trajectories on patients’ outcome and mortality.

Our results confirm that sustained hypotension is strongly associated with unfavourable outcome and mortality. Hypotension can be difficult to control in the pre-hospital setting, especially if related to active bleeding and its occurrence is associated with a higher number of sustained events and poor outcomes [23]. Hypoxia could be potentially more easily recognized and managed even at the scene of the accident through ventilation and orotracheal intubation, thus explaining why the rate of sustained hypoxia in our population was low (2%).

Both hypoxia and hypotension can affect cerebral perfusion pressure, resulting in brain-tissue ischaemia, further cytotoxic oedema and poor outcome [24]. In our cohort, we found few new events for both hypoxia and hypotension, likely because secondary injuries were detected early and effectively treated in the pre-hospital setting. The persistence of hypoxia and hypotension was due to concomitant extracranial injuries in polytrauma patients. Systemic bleeding, which determines systemic hypotension, was associated with poor outcome and increased mortality. Similarly, sustained hypoxia was associated with concomitant chest trauma and difficult oxygenation. However, a resolved hypoxia at the scene [25–28] was associated with better outcomes compared to a resolved hypotension.

Pupillary changes presented a different pattern: not only the sustained event, but even the occurrence of pupillary changes as new events were strongly associated with a poor outcome. This is probably explained because dilated or unreactive pupils are late signs of high intracranial pressure that currently cannot be detected with established monitoring tools and targeted therapies in the pre-hospital settings. No association between the trajectory of pupillary changes and extracranial lesions was found. Although profound hypotension and respiratory derangement with consequent hypercapnia can determinate cerebral swelling, pupillary changes are strictly linked with the severity of TBI itself, and probably early haemodynamic management and airway control is not always sufficient to treat the neurological injury.

Therefore, our results suggest that the reversibility of a systemic insult that occurred in the early post-TBI phase is associated with an improvement of patients’ outcomes. We believe that a prompt recognition of these insults and their aggressive treatment in the pre-hospital setting is mandatory, although it has not been formally proven yet to be effective [29, 30].

Several limitations in our study need to be mentioned. First, this is a retrospective and single centre study, reflecting our own out-of-hospital management. Therefore, with caution, generalization of the results to all TBI cases could be done. Second, there are some missing data in the database and in the clinical records, including information about the mechanism of trauma. Third, we did not analyse the details of the clinical management in the pre-hospital and hospital setting and how each intervention (ventilator parameters -inspired fraction of oxygen, positive end expiratory pressure, tidal volume-, use and type of fluids or vasopressors, osmotic therapies) modified the insults. However, as described in the methods section, treatment at the scene closely followed ALS and ATLS protocols and it should be noted that, in the emergency setting, despite existing treatment protocols, the clinician often decides on the procedures and therapies to fix deranged parameters based on the personal experience. Furthermore, the aim of our study was to understand if the resolution of an event corresponded to an improved outcome, regardless of how it was achieved.

## Conclusion

Early trajectories of secondary injuries can be useful to define their evolution and to better understand how early emergency treatment could impact patients’ outcomes after TBI. We found that the pre-hospital treatment and resolution of systemic physiological derangement were associated with improved outcomes. Sustained hypotension was the strongest determinant of a negative outcome. Further prospective studies using standardized treatment protocols will be needed to confirm our findings.

## Abbreviations

ALS: Advance Life Support
ATLS: Advanced Trauma Life Support
AUC: Area Under the Receiver Operating Characteristic Curve
CI: Confidence Interval
CT: Computed Tomography
GCS: Glasgow Coma Scale
GOS: Glasgow Outcome Scale
IMPACT: International Mission on Prognosis and Analysis of Clinical Trials
MAP: Mean Arterial Pressure
NICU: Neuro Intensive Care Unit
OR: Odds Ratio
SAH: Subarachnoid Haemorrhage
SBP: Systolic Blood Pressure
SI: Secondary Insults
SpO_2_: pulse oximeter's measure of arterial saturation
TBI: Traumatic Brain Injury
